# Syndecan 2 proteoglycan serves as a hepatitis B virus cell attachment receptor

**DOI:** 10.1128/jvi.00796-25

**Published:** 2025-06-26

**Authors:** Sachin Kumar Tripathi, Yingying Li, Guangxiang Luo

**Affiliations:** 1 Department of Microbiology and Immunology, Wake Forest University School of Medicine 542219, Winston-Salem, North Carolina, USA; 2 Department of Microbiology, University of Alabama at Birmingham School of Medicine 318277, Birmingham, Alabama, USA; The University of Arizona, Tucson, Arizona, USA

**Keywords:** entry, infection, syndecan 2, attachment receptor, hepatitis B virus (HBV)

## Abstract

We have previously found that human apolipoprotein E (apoE) is enriched on the envelope of infectious hepatitis B virus (HBV) and plays an important role in HBV infection and morphogenesis. Recently, we have demonstrated that the low-density lipoprotein receptor (LDLR) is required for efficient HBV infection. LDLR is a known apoE-binding receptor. Additionally, heparan sulfate proteoglycans (HSPGs) serve as apoE-binding receptors. HSPGs are implicated in HBV infection as HBV cell attachment receptors. HSPGs are composed of heparan sulfate glycosaminoglycans covalently attached to core proteins, including syndecans (SDC1–SDC4) and glypicans (GPC1–GPC6). GPC5 was previously reported as an HBV entry-promoting factor. In the present study, we have identified SDC2 as another cell attachment receptor promoting HBV infection. Small interfering RNA (siRNA)-induced silencing of SDC2 expression resulted in a significant reduction of HBV infection. Likewise, SDC2 gene knockout decreased the susceptibility of hepatocytes to HBV infection. However, the defective HBV infection in the SDC2-deficient hepatocytes could be fully restored by ectopic SDC2 expression. The importance of SDC2 in HBV infection was validated using primary human hepatocytes. Moreover, SDC2 deficiency lowered preS1- and apoE-binding and consequently HBV attachment to the surface of hepatocytes. Collectively, our findings suggest that SDC2 functions as an HBV cell attachment receptor.

**IMPORTANCE:**

Many different DNA and RNA viruses use HSPGs as cell attachment receptors. HSPGs are composed of core proteins and covalently attached heparan sulfate glycosaminoglycans. Individual SDCs and GPCs play distinct roles in the mediation of cell attachment of different viruses. GPC5 was previously found to promote HBV infection. However, the role of SDCs in HBV infection has not been experimentally examined. In the present study, we have identified SDC2 as an HBV cell attachment receptor. We further found that SDC2-deficient hepatocytes are much less susceptible to preS1- and apoE-binding. These findings suggest that SDC2 promote HBV infection likely through interactions with apoE and preS1, both of which are present on the surface of HBV envelope and contain HSPG-binding sites.

## INTRODUCTION

Hepatitis B virus (HBV) infection continues to pose a major threat to global public health by chronically infecting hundreds of million people worldwide. HBV is a leading cause of common liver diseases such as chronic hepatitis B, cirrhosis, and hepatocellular carcinoma (HCC) (1). Although licensed hepatitis B vaccines are effective to prevent new HBV infection, they do not provide therapeutic benefit to those individuals already infected with HBV. Current antiviral therapies consisting of interferon (IFN) and nucleoside/nucleotide derivatives are efficacious to suppress HBV replication but do not eliminate HBV for most chronic hepatitis B patients (2).

HBV is an enveloped DNA virus belonging to *Hepadnaviridae* family. It has a partially double-stranded circular DNA genome that is converted from a terminally redundant pregenomic RNA (pgRNA) inside viral capsids. PgRNA is encapsidated by core protein together with the viral polymerase (pol), which is a reverse transcriptase and is also referred terminal protein (TP). The envelope of HBV virions contains three different forms (large, middle, and small) of viral surface protein (HBsAg), L-HBsAg, M-HBsAg, and S-HBsAg (3, 4). HBV enters cells through receptor-mediated endocytosis. The sodium taurocholate cotransporting polypeptide (NTCP) is a key receptor essential for HBV infection (5). Upon HBV internalization and uncoating, TP is removed from the relaxed circular DNA (rcDNA) genome (6, 7). The deproteinized rcDNA (DP-rcDNA) is transported to the nucleus and is subsequently converted to a covalently closed circular DNA (cccDNA). The rcDNA to cccDNA conversion is catalyzed by cellular enzymes, including DNA polymerase, endonucleases, and ligase (8–10). The cccDNA acts as the template for transcription of all viral RNAs by the cellular Pol II polymerase, including pgRNA and mRNAs, which encode precore (preC), core (HBcAg), pol, L/M/S-HBsAg, and X protein (HBx) (3). The preC is proteolytically cleaved at both the N- and C-termini by cellular proteases, producing a secreted HBeAg that is considered a viral marker of HBV DNA replication (11). The viral nucleocapsid containing rcDNA and pol is enveloped with HBsAg to produce progeny virions that egress from infected cells. Alternatively, the rcDNA-containing capsids in the cytoplasm are imported back to the nucleus for cccDNA synthesis (3). The HBV infectious cycle requires orchestrated engagement between viral components and many different cellular proteins.

HBV infects hepatocytes by binding to cell surface receptors, such as NTCP and other cell entry-promoting factors. Several previous studies suggested that heparan sulfate proteoglycans (HSPGs) mediate HBV cell attachment (12–14). Heparin was able to inhibit HBV infection in cell culture (14). Likewise, removal of HSPG from cell surface by treatment with heparinase reduced the susceptibility of hepatocytes to HBV infection (13). HSPGs consist of core proteins and covalently attached heparan sulfate (HS) glycosaminoglycans. HSPG core proteins include the membrane-spanning syndecans (SDCs), lycosylphosphatidylinositol-linked glypicans (GPCs), basement membrane proteoglycan perlecan (HSPG2), and agrin (15, 16). One of the HSPG core proteins, GPC5, was previously shown to promote HBV infection (17). In general, human viruses use different HSPGs for their initial cell attachment. For instance, dengue virus uses SDC2 proteoglycan as its binding receptor (18). Human immunodeficiency virus type 1 (HIV-1) prefers SDC3-containing HSPG as the major attachment receptor on dendritic cells (DCs) although SDC1, SDC2, and SDC4 can also serve as HIV-1 attachment receptors when ectopically overexpressed in nonpermissive cell types (19). SDC1 was identified as a cell attachment receptor for both hepatitis C virus (HCV) and human papillomavirus (HPV) (20, 21). Clearly, various HSPGs play distinct roles in the infection of many different viruses.

In addition to HSPGs, HBV also uses the low-density lipoprotein receptor (LDLR) for its cell attachment, as determined by our recent work (22). It is known that both HSPGs and LDLR serve as apolipoprotein E (apoE)-binding receptors, which play pivotal roles in lipid and cholesterol metabolism (22). We have previously demonstrated that human apoE is enriched on the envelope of infectious HBV and plays an important role in HBV infection and morphogenesis (23). Therefore, it is conceivable that apoE on the HBV envelope can mediate HBV cell attachment by binding to cell surface HSPGs and LDLR family receptors. In the present study, we have further identified SDC2 as a cell attachment receptor promoting HBV infection. Silencing of SDC2 expression and SDC2 gene knockout significantly reduced HBV infection. However, the defective HBV infection in the SDC2-deficient hepatocytes could be fully restored by ectopic SDC2 expression. More significantly, SDC2 deficiency impaired preS1- and apoE-binding capacity of hepatocytes and consequently HBV cell attachment. These findings suggest that SDC2 serves as an HBV cell attachment receptor probably via interaction with both apoE and L-HBsAg on the HBV envelope.

## RESULTS

### Profiling of syndecans in HBV infection

**Fig 1:**
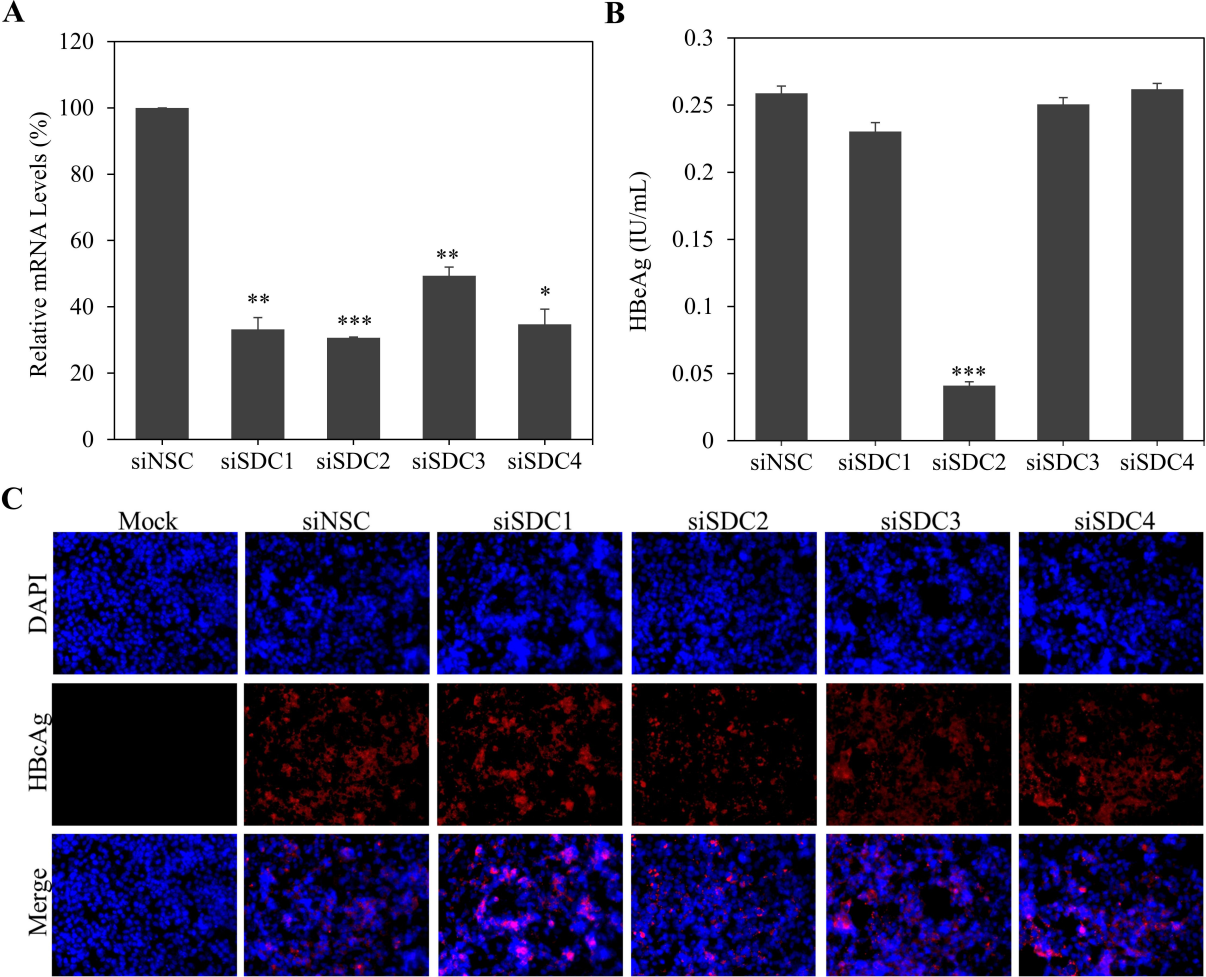
Effects of siRNA-medicated knockdown of syndecans on HBV infection. HepG2^NTCP^ cells of 1.5 × 10^5^/well were subjected to transfection with 50 nM of SDC1, SDC2, SDC3, or SDC4 siRNAs using RNAiMax reagent. At 48 h post transfection (p.t.), cells were infected with HBV at 37°C for 12 h. Upon removal of unbound HBV, cells were incubated with DME/F12 medium containing 3% FBS, 1% DMSO, and 5 µg/mL hydrocortisone. After 4 days p.i., total RNA was extracted. The levels of SDC1, SDC2, SDC3, and SDC4 mRNAs were quantified by a qRT-PCR method (**A**). The levels of HBeAg in cell culture supernatants were determined using a chemiluminescence immunoassay (**B**). The average levels of HBeAg were calculated from three independent experiments. HBcAg expression in the HBV-infected HepG2^NTCP^ cells was detected by IFA (**C**). *P < 0.05, **P < 0.01, ***P < 0.001.

**Fig 2:**
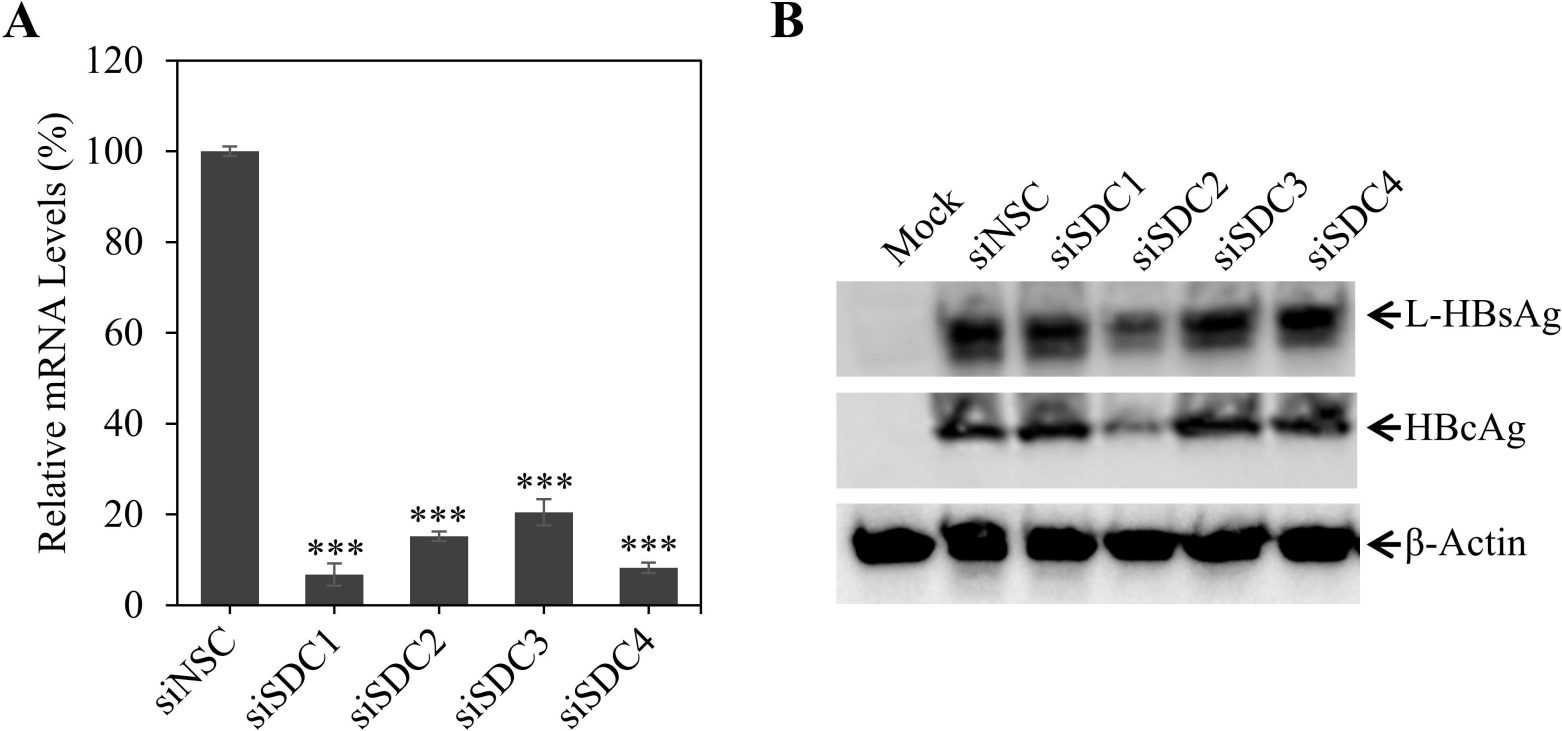
Validation of SDC2 important for HBV infection of primary human hepatocytes (PHHs). PHHs in a 24-well plate were transfected with 100 nM of SDC1, SDC2, SDC3, or SDC4 siRNAs using RNAiMax reagent. HBV infection, total RNA extraction, and RT-PCR quantification of SDC1, SDC2, SDC3, and SDC4 mRNAs were the same as described in Fig. 1. (**A**) siRNA-induced degradation of SDC mRNAs. The average levels of SDC1, SDC2, SDC3, and SDC3 mRNAs from triplicate repeats are shown as percentage relative to that of a nonspecific siRNA-transfected cells. ***P < 0.001. (**B**) The levels of L-HBsAg and HBcAg in the HBV-infected PHHs determined by Western blot using a PreS1- and HBcAg-specific monoclonal antibodies 7H11 and T2221, respectively.

There are four different human syndecans, SDC1, SDC2, SDC3, and SDC4, which are implicated in the infection of many different viruses. Our previous studies found that both SDC1 and SDC2 play important roles in hepatitis C virus (HCV) infection and cell-to-cell spread (20, 24). To determine whether any specific SDC is important for HBV infection, we profiled all four SDCs using SDC-specific small interfering RNAs (siRNAs), which consist of four siRNAs (SmartPool) targeting different regions of each mRNA, to silence the expression of individual SDCs. These siRNAs were previously shown to lower the levels of SDC mRNAs by greater than 90% in Huh-7 cells (20). They were less potent in HepG2^NTCP^ cells and reduced the levels of each SDC mRNA by 50%–70% (Fig. 1A). The siRNA-transfected HepG2^NTCP^ cells were then infected with HBV at 48 h after siRNA transfection. The efficiency of HBV infection was determined by the levels of HBcAg in the cell and HBeAg in cell culture supernatants. The levels of HBeAg in the supernatants were lowered by about 80% when SDC2 expression was silenced in HepG2^NTCP^ cells (Fig. 1B). Likewise, HBcAg in the HBV-infected cells were also significantly decreased, as shown by immunofluorescences assay (Fig. 1C). These findings were further validated using primary human hepatocytes (PHHs). Each of the SDC-specific siRNAs resulted in 80%–90% reduction of their corresponding mRNAs (Fig. 2A). However, only SDC2 knockdown expression significantly decreased HBV infection, as shown by the two fold reduction of L-HBsAg and HBcAg, respectively (Fig. 2B). Taken together, these results suggest that SDC2 plays an important role in HBV infection.

### Importance of SDC2 for HBV infection

**Fig 3:**
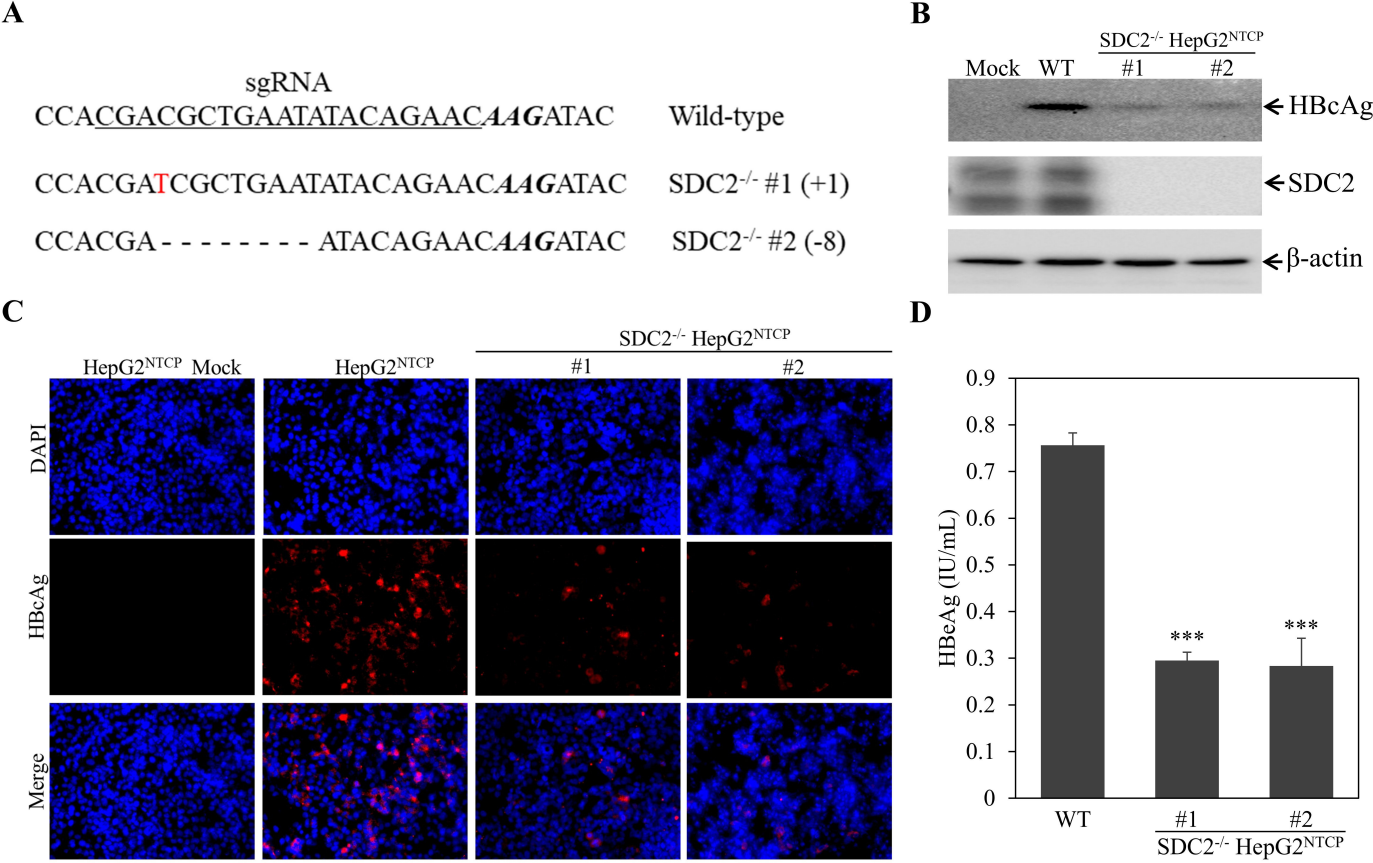
Reduction of HBV infection in SDC2-deficient HepG2^NTCP^ cells. Stable SDC2-knockout (SDC2^-/-^) HepG2^NTCP^ cell lines were obtained as described in the Materials and Methods. SDC2 deficiency in stable cell lines were confirmed by DNA sequencing (**A**). The SDC2-deficient cell lines were infected with HBV in a parallel comparison with parent HepG2^NTCP^ (wild type, WT). At 4 days p.i., the levels of SDC2 and HBcAg were determined by Western blot analysis using β-actin as a house-keeping gene control (**B**). The effects of SDC2 deficiency on HBV infection were also determined by IFA for HBcAg-staining in the HBV-infected cells (**C**) as well as the levels of HBeAg in the cell culture supernatants (**D**). The average levels of HBeAg were calculated from triplicate repeats. ***P < 0.001.

To further determine the importance of SDC2 in HBV infection, we have made SDC2-deficient HepG2^NTCP^ cell lines using CRISPR/Cas9 gene editing system, as described in our previous work (24). We chose two independent SDC2-knockout HepG2^NTCP^ cell clones for HBV infection. The knockout of SDC2 gene was validated by DNA sequence analysis (Fig. 3A) and Western blotting (Fig. 3B). Either one nucleotide insertion (cell clone #1) or eight-nucleotide deletion (cell clone #2) resulted in a disruption of the open reading frame of SDC2 (Fig. 3A) and the loss of SDC2 expression (Fig. 3B). As a result, SDC2 deficiency reduced the levels of HBcAg in the HBV-infected cells by 73% to 85%, as determined by both Western blot analysis (Fig. 3B) and IFA (Fig. 3C). Additionally, the levels of HBeAg in the cell culture supernatants were lowered by about three fold (Fig. 3D), as quantified by an enzyme-linked immunosorbent assay (ELISA). These results demonstrate that SDC2 plays an important role in HBV infection.

### Restoration of defective HBV infection in SDC2-deficient cells by ectopic SDC2 expression

**Fig 4:**
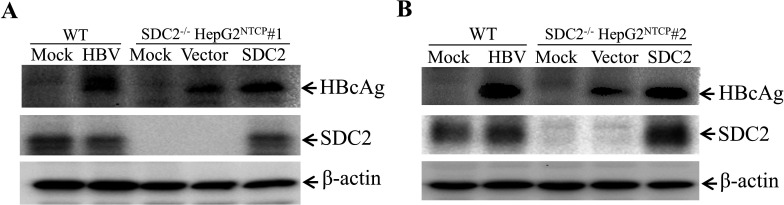
Restoration of defective HBV infection by ectopic SDC2 overexpression. Parental and SDC2^-/-^ HepG2^NTCP^ cell lines seeded in 24-well plates were transfected with an SDC2-expressing plasmid DNA (pCMV6-XL4-hSDC2). At 48 h p.t., cells were infected with HBV in the presence of 4% PEG at 37°C for 12 h. After 4 days p.i., HBV-infected cells were lysed in a RIPA buffer. HBcAg, SDC2, and β-actin in cell lysates were detected by Western blot analysis using antibodies specific to corresponding proteins. (**A**) SDC2^-/-^ cell line #1; (**B**) SDC2^-/-^ cell line #2.

The question arose whether the defective HBV infection in the SDC2-deficient cells was due to possible off-target effect associated with CRISPR/Cas9 gene-editing methodology. To exclude this possibility, a plasmid DNA expressing SDC2 was transfected to SDC2-deficient HepG2^NTCP^ cells prior to HBV infection. The levels of HBcAg in the HBV-infected cells were determined by Western blot analysis. Ectopic expression of SDC2 in both SDC2-deficient cell clones was able to fully restore HBV infection, as shown by similar levels of HBcAg in the HBV-infected cells (Fig. 4). These data further confirm that the defective HBV infection in the SDC2-knockout cells was indeed due to SDC2 deficiency, demonstrating that SDC2 is important for efficient HBV infection.

### Role of SDC2 in HBV cell attachment

**Fig 5:**
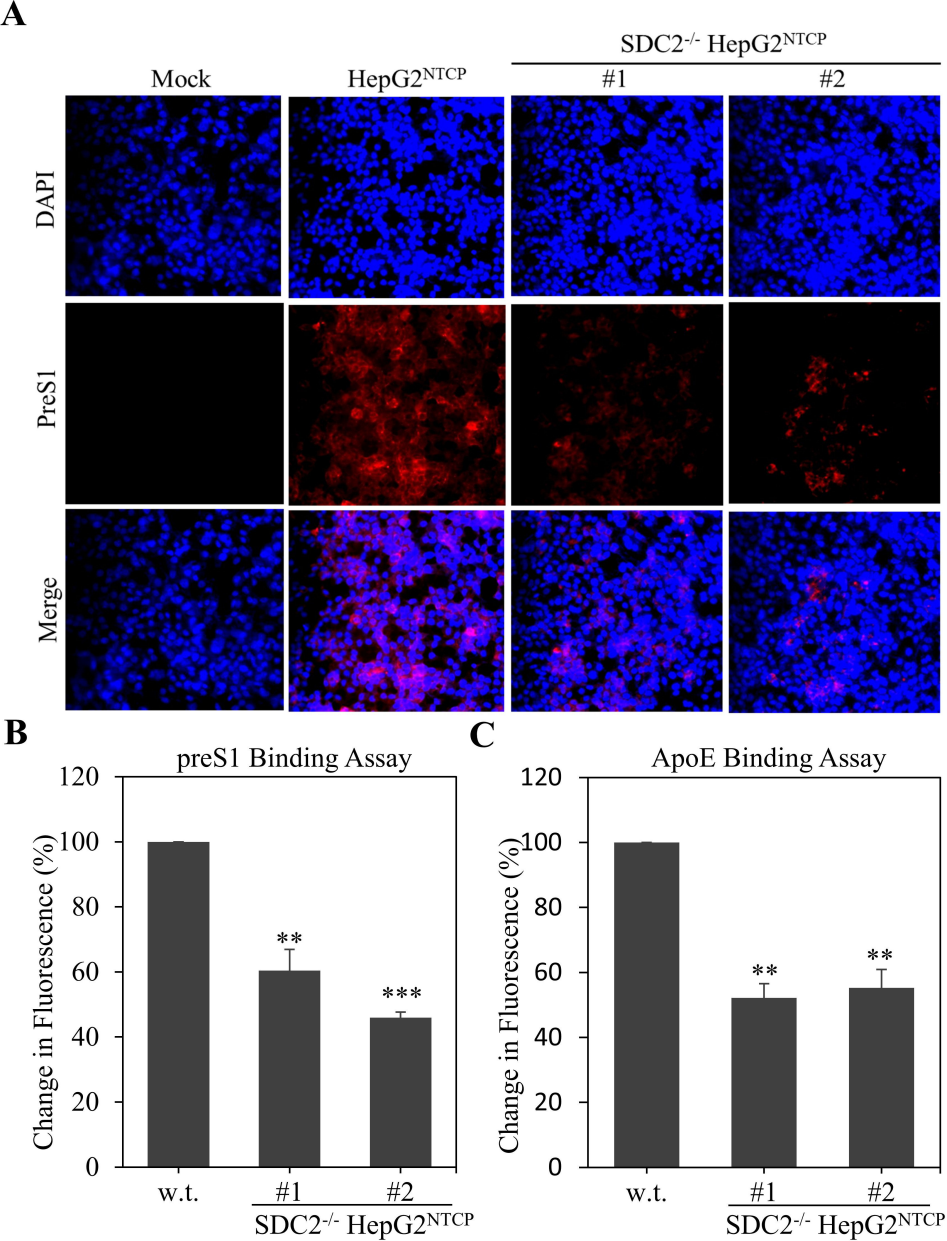
Comparison of preS1 and ApoE peptide binding between parental and SDC2^-/-^HepG2^NTCP^ cells. Parental and SDC2^-/-^ HepG2^NTCP^ cells seeded in a 96-well plate at a density of 3 × 10^4^ cells/well were incubated with Myr-preS1_2-48_ or apoE_131-162_ (40 nM) on ice for 1 h. Unbound peptides were removed by washing with 1× PBS three times. Peptide-bound cells were fixed with 4% paraformaldehyde (PFA). Nuclei were stained with DAPI. Images were visualized under a fluorescence microscope. (**A**) Images of Myr-preS1_2-48_ binding to parental and SDC2^-/-^ HepG2^NTCP^ cells. (**B**) Comparison of Myr-preS1_2-48_ or (C) apoE_131-162_ binding capacity between parent and SDC2^-/-^ HepG2^NTCP^ cells. Fluorescence intensity was quantified with a fluorometer. The average fluorescence intensity was calculated from triplicate repeats. **P < 0.01, ***P < 0.001.

HSPGs are apoE-binding receptors and were also found to bind to preS1 of the L-HBsAg and S-HBsAg as well (12). To further determine the underlying molecular mechanism of SDC2 in HBV infection, we made TAMRA-conjugated preS1 and apoE peptides to directly compare the differences in preS1- and apoE peptide binding between parental and SDC2-deficient HepG2^NTCP^ cells. The preS1 peptide consists of the amino acid residues 2–48 that is N-terminally myristoylated and C-terminally conjugated with TAMRA. This preS1 peptide was previously used by others to probe interactions between preS1 and NTCP or preS1 and HSPGs (25, 26). The apoE peptide covers the receptor binding pocket from amino acids 131–162 with an extra myristoylated C-terminal cysteine and N-terminal lysine conjugated to TAMRA. Interestingly, SDC2-deficiency reduced the binding of both preS1- and apoE peptides to HepG2^NTCP^ cells by 40%–60% (Fig. 5). These results suggest that SDC2 likely interacted with both preS1 and apoE on the HBV envelope and consequently promoted HBV cell attachment and infection.

**Fig 6:**
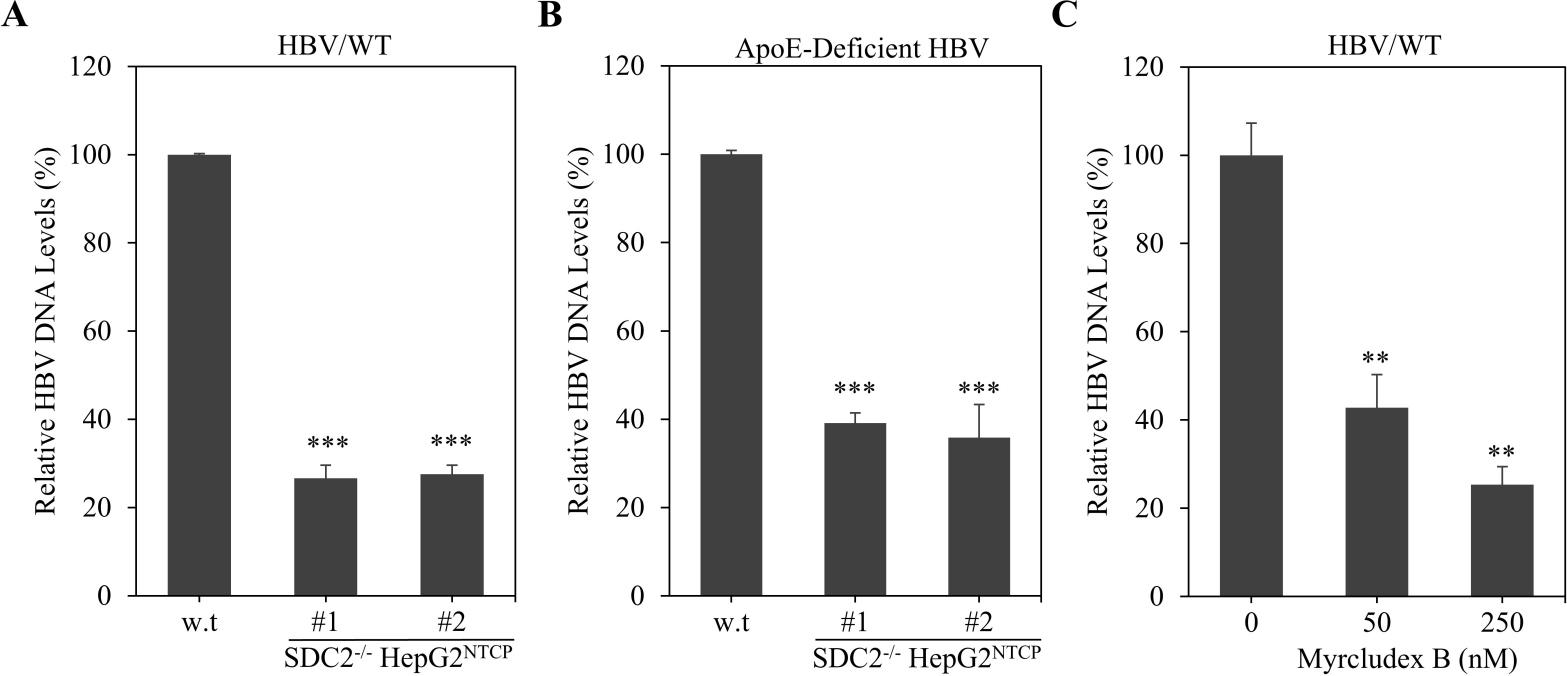
Loss of HBV cell attachment capacity in SDC2^-/-^ HepG2^NTCP^ cells. Parental and SDC2^-/-^ HepG2^NTCP^ cells in 24-well cell culture plates were incubated with HBV grown from HepAD38 cells (**A**) or HBV grown from apoE-deficient (apoE^-/-^) HepAD38 cells (**B**) at 37°C for 6 h. Unbound HBV was removed by washing with 1xPBS three times. HBV DNA extracted from cells was quantified by a qPCR method. To examine inhibition of HBV attachment by preS1 peptide, HepG2^NTCP^ cells were incubated with HBV in the presence of varying concentrations of Myrcludex B (0, 50, and 250 nM) at 37°C for 6 h (**C**). Unbound HBV was removed by washing with 1× PBS three times. HBV DNA extracted from HBV-infected cells was quantified by a qPCR method. Relative HBV DNA levels as percentage of control are plotted. The average HBV DNA levels was calculated from triplicate repeats. **P < 0.01, ***P < 0.001.

To further validate the role of SDC2 in HBV cell attachment, we carried out a direct HBV attachment assay. Parental and SDC2-deficient HepG2^NTCP^ cells were incubated with HBV for 6 h. Unbound HBV was removed by extensive washing with PBS. Viral DNA was extracted from HBV-infected cells and was quantified by a real-time PCR method. Like preS1 and apoE peptide binding, SDC2 deficiency also lowered the levels of HBV DNA by about 80% (Fig. 6A). We also test cell attachment of HBV grown from apoE-knockout HepAD38 cells, as described in our previous work (23). Interestingly, SDC2 deficiency reduced the cell attachment of an apoE-deficient HBV by about 60%, suggesting that the interaction between SDC2 and S-HBsAg/L-HBsAg was impaired (Fig. 6B). To further confirm the preS1-mediated HBV attachment, an N-terminally myristoylated preS1 peptide (Myrcludex B) was added during HBV attachment. Indeed, it inhibited HBV attachment by about 60%–75% in a dose-dependent manner (Fig. 6C). These results suggest that SDC2 proteoglycans mediate HBV cell attachment probably through interaction with both apoE, preS1 of the L-HBsAg, and S-HBsAg on the HBV virions.

## DISCUSSION

Cell surface molecules mediating virus attachment and entry to target cells are designated as virus receptors, which can be divided into two different groups, attachment and post-attachment receptors (24). Attachment receptors facilitate virus binding to susceptible cells, which are also referred virus entry promoting factors. Post-attachment receptors/coreceptors mediate virus internalization and/or fusion between viral and cellular membranes, resulting in the release of viral genome to the cytosol or nucleus for replication. Viral attachment and cell entry can be mediated by either a single receptor or multiple different receptors/co-receptors. In the case of HBV, NTCP is a key receptor essential for HBV infection (5), although several other host factors were identified as attachment receptors or entry cofactors promoting HBV infection (12, 22, 26, 27). We have previously found that LDLR promotes HBV infection by interacting with the host apoE enriched on the envelope of HBV virions (22). Epidermal growth factor receptor (EGFR) was also reported as a host cofactor mediating HBV internalization probably via its interactions with NTCP (25). Additionally, GPC5, one of the HSPG core proteins was shown to promote HBV infection (17).

In the present study, we have obtained substantial evidence to demonstrate that SDC2 plays an important role in HBV infection. Downregulation of SDC2 expression by siRNA-induced degradation of SDC2 mRNA significantly reduced HBV infection in both HepG2^NTCP^ cells and PHHs (Fig. 1 and 2). Likewise, knockout of SDC2 gene by CRISPR/Cas9-induced gene editing technique resulted in about 80% reduction of HBV infection (Fig. 3). The defective HBV infection could be fully restored by ectopic SDC2 expression among SDC2-deficient cells (Fig. 4). Additionally, SDC2 deficiency lowered the cell binding of both preS1 and apoE peptides (Fig. 5). More importantly, SDC2 deficiency reduced HBV cell attachment in both parental and SDC2-knockout HepG2^NTCP^ cells (Fig. 6A and B). A synthetic preS1 peptide was able to block HBV attachment (Fig. 6C). Taken together, these findings suggest that SDC2 proteoglycans serve as HBV attachment receptors, consistent with the important role of HSPGs in the mediation of HBV infection, as previously reported (12–14, 17). SDC2 proteoglycan is one of the HSPG family receptors that are composed of many different members, including the previously reported GPC5 proteoglycan as an HBV entry-promoting factor (17). Additionally, other host factors are implicated in HBV cell attachment, including the LDLR family receptors and endothelial lipase (22, 26). Therefore, silencing or knockout of these redundant host genes individually will unlikely ablate HBV infection. This may explain why SDC2 gene knockout did not eliminate HBV cell attachment (Fig. 6). Future investigations are warranted to validate the physiological importance of SDC2 and other HBV cell attachment receptors in HBV infection *in vivo* using a fully humanized mouse model.

How SDC2 proteoglycan mediates HBV cell attachment remains enigmatic. The HS glycosaminoglycans attached to SDC2 can bind to apoE, preS1 of the L-HBsAg, and S-HBsAg on the HBV envelope via electrostatic interactions, as suggested by significantly reduced preS1 and apoE peptide-binding capacity of the SDC2-deficient HepG2^NTCP^ cells (Fig. 5). PreS1 has two separate NTCP- and HSPGs-binding sites next to each other within the N-terminal 48 amino acid residues (28). Another HS-binding site was found in the antigenic loop of the S-HBsAg with two positively charged residues R122 and K141 critical for HS binding (12). These two HS-binding sites in the preS1 and S-HBsAg may mediate HBV attachment to SDC2 proteoglycan on the surface of hepatocyte. Additionally, we have previously demonstrated that human apoE is enriched on the envelope of HBV and HCV and plays an important role in both HBV and HCV infections (23, 29). The HBV-associated apoE can bind to both HSPGs and LDLR, two major apoE-binding receptors ubiquitously present in many different cell types. LDLR is also required for efficient HBV infection as determined by our previous studies (22). ApoE is believed to interact with the cysteine-rich repeat regions of the LDLR family members and the 2-O-sulfo groups of the iduronic acid monosaccharides or the N- and 6-O-sulfo groups of the glucosamine sulfate monosaccharides of HSPGs (30). Interestingly, the *O*-sulfonated heparin at physiological concentrations was found to enhance PEG-independent HBV infection more efficiently compared to N-sulfonated heparin (31). Collectively, SDC2 proteoglycan mediate HBV cell attachment likely via its interactions with preS1, S-HBsAg, and apoE, which are present on the HBV envelope. It will be interesting to see if HS derivatives will block HBV infection. It was previously reported that 3-*O*-sulfonated HS octasaccharides could inhibit the infection of herpes simplex virus type 1 (HSV-1), which uses 3-O-sulfonated HSPG for its cell attachment (32).

It is not clear why SDC2 but no other SDCs is involved in HBV cell attachment. The function of SDCs in viral infection is likely determined by multiple factors. The levels of individual SDC expression in hepatocytes vary. It appeared that the levels of SDC2 expression in HepG2^NTCP^ and Huh-7^NTCP^ are the highest compared with SDC1, SDC2, and SDC3 (Tripathi and Luo, unpublished data). HS attachment is also critically important for the function of SDCs (33). There are three highly conserved sites for HS attachment at the N-terminal ectodomain of SDCs. HS attachment to each of the three serine residues individually or in different combinations will likely influence their preferential binding to different ligands on the viral envelopes. More significantly, sulfonation of heparin glucosamine is key for ligand selection. For instance, HSV-1 preferentially uses the 3-O-sulfonated heparin glucosamine as its cell attachment receptor (32). Future studies are warranted to further determine the molecular basis underlying the role of SDC2 and other members of HSPGs in HBV cell attachment and infection.

## MATERIALS AND METHODS

### Cell culture

The NTCP-expressing HepG2 cells (HepG2^NTCP^), primary human hepatocytes (PHHs), and HBV-producing HepAD38 cells were described previously (23). HepG2^NTCP^ and HepAD38 cells were maintained in DME/F12 medium supplemented with 10% fetal bovine serum (Gemini), 100 U/mL penicillin, 100 U/mL streptomycin (Corning), 1× MEM non-essential amino acids (Corning), and 1× sodium pyruvate (Corning) at 37°C in a 5% CO_₂_ incubator. HepG2^NTCP^ cells were grown in the presence of 2 µg/mL puromycin (Invivogen), and HepAD38 was maintained in 250 µg/mL of G418 (Sigma). PHHs were grown in complete hepatocyte culture medium (Lonza). Cell culture flasks and 24-well plates were coated with 50 µg/mL of rat tail collagen type I (Corning, 354236).

### Antibodies and reagents

HBcAg-specific antibodies T2221 (Institute of Immunology Co., LTD., Tokyo) and C1-5 (Santa Cruz, sc-23945) were used to detect HBcAg. HBV PreS1 monoclonal antibody (7H11) was kindly provided by Shuping Tong (Brown University). SDC2 (sc-365624) was purchased from Santa Cruz. HRP-conjugated goat anti-mouse antibody was obtained from Cell Signaling. Human β-actin monoclonal antibody (AC15) was purchased from Sigma-Aldrich. Protein quantification was performed using Pierce Rapid Gold BCA Protein Assay reagents (Thermo Scientific, A53225). SDC-specific siRNAs and a nonspecific control (NSC) siRNA were synthesized by Horizon Discovery. HBeAg immunoassay (ELISA) kits were purchased from International immunodiagnostics (USA). RNAiMax and DMRIE-C reagents for siRNA and DNA transfection were from Invitrogen. DNA isolation kits (DNeasy Blood and Tissue, Cat No. 69506) were purchased from Qiagen. Clarity Max^TM^ Western blotting ECL substrate were from Bio-Rad. The human SDC2-expressing plasmid pCMV6-XL4/SDC2 were obtained from OriGene.

### HBV production and concentration

HepAD38 cells were grown in DME/F12 medium containing 3% tetracycline negative FBS (Gemini) and 1% dimethyl sulfoxide (DMSO) (Sigma) in HyperFlasks (Corning) as described previously (22). Cell culture supernatants were collected every 3 days and were passed through 0.45 µm membrane filter. HBV was concentrated using Amicon Ultra Centrifugal Filter (Millipore Sigma, UFC910096). The genome copy numbers of HBV DNA were quantified by a real-time PCR method using the previously described primers and probe (22, 23).

### HBV infection

HepG2^NTCP^ cells were seeded in 24-well cell culture plates at a density of 2 × 10⁵ per well overnight and infected with HBV at a multiplicity of infection (m.o.i.) of 100–500 genome equivalent copies in the presence of 4% PEG 8000 (Sigma) as described previously (22, 23, 34). After 12 h of infection, unbound HBV was removed by extensive washing with 1× PBS. The HBV-infected cells were then incubated at 37°C for 4 days in DME/F12 medium supplemented with 3% FBS, 1% DMSO, and 5 µg/mL hydrocortisone (HC). The levels of HBcAg in the cell were determined by Western blot and/or immunofluorescence assay (IFA). The levels of HBeAg in the supernatants were quantified by ELISA.

### siRNA transfection

SDC-specific SmartPool siRNAs or a nonspecific control siRNA (siNSC) were transfected into HepG2^NTCP^ cells and PHHs using lipofectamine RNAiMax reagent. At 48 h post-transfection (p.t.), cells were infected with HBV in the presence of 4% PEG at 37°C for 12 h. After 4 days p.i., the levels of HBcAg and HBeAg in the cell and supernatant were determined, respectively.

### Construction of SDC2 knockout cell lines

The method for construction of a recombinant lentivirus expressing SDC2 gene-specific sgRNA was described in our previous work (20, 24). HepG2^NTCP^ cells in 100 mm cell culture dishes were transduced with lentiCRISPRv2-blasticidin lentivirus expressing SDC-2 sgRNA. Stable cell clones were selected in the presence of 5 µg/mL blasticidin for 2–3 weeks. Individual cell clones were transferred to 24-well cell culture plates for expansion. Cellular DNAs were extracted from each cell clone. SDC2 DNA was amplified by PCR using SDC2-F (5′-AGACTACCCACAGACACC-3′) and SDC2-R (5′-AGACTAGGACCACCTCAA-3′) as primers and was subject to DNA sequence analysis. The knockout of SDC2 gene in stable cell clones was further confirmed by Western blot analysis.

### Ectopic SDC2 expression

Parental and SDC2^⁻/⁻^ HepG2^NTCP^ cells were seeded at a density of 1.5 × 10^5^ cells per well in 24-well cell culture plates. Cells were transfected with a plasmid DNA expressing SDC2 (pCMV6-XL4/SDC2) using DMRIE-C reagent. After 48 h p.t., cells were infected with HBV as described above. After 4 days p.i., the levels of HBcAg were determined by Western blot.

### Real-time quantitative polymerase chain reaction (qPCR)

HBV DNA was quantified by qPCR using two HBV-specific primers: 5′-GAGTGTGGATTCGCACTCC-3′ (forward) and 5′-GAGGCGAGGGAGTTCTTCT-3′ (reverse) and probe (5′-FAM-CCGTGTGCACTTCGCTTCA CCTCTGC-TAMRA-3′), as described previously (22, 23). The levels of SDC and β-actin mRNAs were determined by reverse transcription (RT) qPCR method using specific primers as described in our previous work (20). cDNAs were synthesized from total RNAs using AMV reverse transcriptase (New England Biolabs), followed by qPCR. qPCR was carried out as follows: 10 min at 95°C and 40 cycles at 95°C for 15 s and 60°C for 1 min using Power Sybr Green Master Mix (Applied Biosystems). β-actin was used as a house-keeping gene control to normalize total amounts of RNAs used in qRT-PCR.

### Quantification of HBeAg by chemiluminescence immunoassay

The levels of HBeAg in the cell culture supernatants were measured using an ELISA (International Immunodiagnostics) following the manufacturer’s instructions, as previously described (22, 23). Absorbance was measured at 450 nm using a plate reader (BioTek).

### Western blotting analysis

HepG2^NTCP^ cells were lysed in RIPA buffer. Protein concentrations of cell lysates were quantified using BCA protein assay reagent. A total of 25 µg of protein was loaded onto a 4%–20% SDS-PAGE gel. Following electrophoresis, proteins were transferred onto a polyvinylidene difluoride (PVDF) membrane using a semidry blotting system (Bio-Rad). Protein membrane was blocked with 5% nonfat dry milk and incubated with primary antibodies specific to HBcAg, SDC2, and β-actin at 4°C overnight. Horseradish peroxidase (HRP)-conjugated secondary antibodies were incubated at room temperature for 1 h, followed by incubation with an ECL substrate (Bio-Rad). Protein bands were visualized and documented using the ChemiDoc MP Imaging System (Bio-Rad).

### Immunofluorescence assay (IFA)

HepG2^NTCP^ cells were seeded at 2 × 10^5^ cells/well in 24-well cell culture plates with coverslips or 1 × 10^5^/well in μ-Slide 8 Well ibiTreat coverslip (ibidi USA, Cat.No:80807) coated with collagen. Cells were infected with HBV as described above. At 4–5 days p.i., HBV-infected cells were fixed with 4% paraformaldehyde at room temperature for 20 min, followed by cell permeabilization with 0.1% Triton X-100 for 10 min. Cells were incubated with a blocking solution (3% BSA) at 4°C overnight. HBcAg was stained with a HBc-specific monoclonal antibody C1-5 (100-fold dilution) at 4°C overnight and the secondary goat anti-mouse IgG conjugated with Alexa Flur 594 (2,000-fold dilution) (Thermofisher) at room temperature for 1 h. Nuclei were stained with DAPI (1 µg/mL) for 10 min. Images were visualized using a fluorescence microscope or confocal (Leica, STELLARIS), which were processed by Image J.

### PreS1 and apoE peptide binding

A preS1 peptide consisting of amino acids 2 to 48 (Myr-GQNLSTSNPLGFFPDHQLDPAFRANTANPDWDFNPNKDTWPDANKVGK-TAMRA) was myristoylated at the N-terminus and TAMRA-conjugated at the C-terminus, as previously described (25, 26). The preS1 is designated Myr-preS1_2-48_. An apoE peptide corresponding to the receptor-binding pocket from amino acids 131 to 162 (CEELRVRLASHLRKLRKRLLRDADDL QKRLAVYK-TAMRA) was C-terminally conjugated to TAMRA, which is named apoE_131-162_. Both Myr-preS1_2-48_ and apoE_131-162_ peptides were synthesized by China Peptides (Shanghai, China). PreS1-mediated attachment to host cells was evaluated by incubating HepG2^NTCP^ cells with 40 nM of either Myr-preS1_2-48_ or apoE_131-162_ peptide on ice for 1 h. Upon extensive washing with 1× PBS, cells were fixed with 4% paraformaldehyde for 20 min. Cell nuclei were stained with DAPI for 10 min. Images were visualized under a fluorescence microscope, and fluorescence intensity was measured with a plate reader (BMG Labtech CLARIOstar Plus) (25).

### HBV cell attachment

Parental and SDC2^⁻/⁻^ HepG2^NTCP^ cells were incubated with HBV at an m.o.i. of approximately 100 genome equivalent copies in DME/F12 medium at 37°C for 6 h (22). Unbound HBV was removed by washing with 1× PBS. Cell-bound HBV DNA was then extracted using a QIAGEN DNA isolation kit as per manufacturer protocol and quantified by a qPCR method.

### Statistical analysis

Statistical analyses were conducted using GraphPad Prism five software. Data were expressed as means ± standard deviations (SD) calculated from triplicates. Comparisons between groups were performed using paired two-tailed *t*-tests and one-way analysis of variance (ANOVA) for multiple comparisons. A *P-*value of less than 0.05 (*P* < 0.05) was considered statistically significant.
